# Fournier’s Gangrene Mortality Index (FGMI): A New Scoring System for Predicting Fournier’s Gangrene Mortality

**DOI:** 10.3390/diagnostics14232732

**Published:** 2024-12-05

**Authors:** Hüseyin Yönder, Mehmet Çelik, Mehmet Sait Berhuni, Ahmed Cihad Genç, Hasan Elkan, Faik Tatlı, Abdullah Özgönül, Felat Çiftçi, Fırat Erkmen, Oğuz Karabay, Ali Uzunköy

**Affiliations:** 1 Department of General Surgery, Faculty of Medicine, Harran University, Şanlıurfa 63300, Turkey; drmsaitberhuni@hotmail.com (M.S.B.); dr_elkan@hotmail.com (H.E.); faiktatli-@hotmail.com (F.T.); drozgonul@yahoo.com (A.Ö.); aliuzunkoy@yahoo.com (A.U.); 2 Department of Infectious Diseases and Clinical Microbiology, Faculty of Medicine, Harran University, Şanlıurfa 63300, Turkey; dr.mcelik12@gmail.com; 3 Department of Internal Medicine, Sakarya Training and Research Hospital, Sakarya 54290, Turkey; genccihad@gmail.com; 4 Clinic of General Surgery, Şanlıurfa Training and Research Hospital, Şanlıurfa 63250, Turkey; felatciftci@gmail.com; 5 Clinic of General Surgery, Şanlıurfa Balıklıgöl State Hospital, Şanlıurfa 63050, Turkey; firaterkmen@gmail.com; 6 Department of Infectious Diseases and Clinical Microbiology, Faculty of Medicine, Sakarya University, Sakarya 54290, Turkey; drkarabay@yahoo.com

**Keywords:** Fournier’s gangrene, LRINEC, Fournier’s gangrene mortality index, predictive

## Abstract

Objectives: Fournier’s gangrene is an aggressive, rapidly progressing, and life-threatening necrotizing fasciitis of the perineal and genital regions. Various scoring systems have been developed for predicting survival and prognosis in Fournier’s gangrene. This retrospective study aimed to evaluate the effectiveness of the newly developed Fournier’s gangrene mortality index (FGMI) in predicting mortality associated with Fournier’s gangrene. Methods: The study included patients over the age of 18 years who were followed-up with a diagnosis of Fournier’s gangrene in the general surgery clinics of three different hospitals in Şanlıurfa province between 2014 and 2024. The patients included in this study were divided into two groups: deceased (*n* = 20) and surviving (*n* = 149). In FGMI, the parameters used were age, creatinine level, albumin level, lymphocyte percentage, and neutrophil-to-lymphocyte ratio. Based on the total score and risk assessment, <5 points were categorized as low-to-moderate mortality risk and ≥5 points as high mortality risk. Results: A total of 169 patients with a diagnosis of Fournier’s gangrene were included in the study; 87 were men (51.48%). The median age of all patients was 53 (40–63) years; 20 patients (11.8%) died. The Laboratory Risk Indicator for Necrotizing Fasciitis (LRINEC) score did not show a statistically significant difference between the deceased and surviving groups (*p* = 0.5). Compared to the survivors, the deceased had higher neutrophil counts, neutrophil percentages, neutrophil-to-lymphocyte ratios, platelet-to-lymphocyte ratios, and C-reactive protein-to-albumin ratios, whereas lymphocyte counts, lymphocyte percentages, eosinophil counts, eosinophil percentages, monocyte counts, and monocyte percentages were lower, and these differences were statistically significant. According to receiver operating characteristic (ROC) analysis, the ROC-area under the curve for predicting mortality based on an FGMI score of ≥5 was 0.88 (95% CI: 0.80–0.95) with a sensitivity of 90% and a specificity of 70% (*p* < 0.001). Univariate risk analysis was performed, and the odds ratio revealed that mortality risk in patients followed-up for Fournier’s gangrene with a FGMI score of ≥5 was 20 times higher (4.48–90.91) (*p* < 0.001). Conclusions: The results reveal that the FGMI score is a scoring system that can predict mortality at the initial clinical presentation of patients with Fournier’s gangrene. Another important finding of the present study is that the LRINEC score was not sufficiently effective in predicting mortality.

## 1. Introduction

Fournier’s gangrene (FG), first described by Alfred Fournier in 1883, is an aggressive, rapidly progressing, and life-threatening necrotizing fasciitis of the perineal and genital regions [1,2]. FG causes obliterative endarteritis, leading to inflammation and edema. Necrosis occurs due to impaired blood circulation in the skin and subcutaneous tissues, leading to purplish-black discoloration of the skin. The hypoxic tissue provides a conducive environment for the growth of anaerobic bacteria, which produce nitrogen and hydrogen gases. Infection can spread along the fascia, leading to necrosis of the perineum, scrotum, lower abdominal wall, and upper thighs [3].

Predisposing factors for FG include diabetes mellitus (DM), urinary incontinence, local trauma, perineal or perirectal surgery, spread of periurethral/anal infections, genitourinary infections, anorectal abscess, immunosuppression, alcoholism, and kidney or liver diseases [4]. Studies show that men, especially in the older age group, are more affected by FG [5,6,7]. Furthermore, FG is typically polymicrobial, and multiple microorganisms are often isolated from wound cultures. Commonly isolated microorganisms include *Staphylococcus* spp., *Streptococcus* spp., *Escherichia coli*, *Pseudomonas* spp., and *Bacteroides* spp., and nonbacterial pathogens such as *Candida* spp. [3,4,5,6,7,8].

The most common clinical symptoms in patients diagnosed with FG are swelling of the external genital organs, pain, and high fever. The average time from the onset of symptoms to hospital admission is 5.1 ± 3.1 days. Moreover, delays in diagnosis after the onset of symptoms can lead to skin necrosis. Erythema can rapidly progress along anatomical fascial planes, with the potential to spread from the perineum to the clavicles along the anterior abdominal wall. Although the involvement of deeper tissues and the testes is rare, it can be a major indicator of a retroperitoneal or intra-abdominal infection source [9]. Due to the high mortality associated with FG, an aggressive treatment approach is required at the initial presentation, including the administration of broad-spectrum antibiotics [3].

Various scoring systems have been developed to predict survival and prognosis in FG. Wong et al. developed the Laboratory Risk Indicator for Necrotizing Fasciitis (LRINEC) scoring system, which is a new, simple, and objective scoring system that can help distinguish necrotizing fasciitis from other soft tissue infections using routine laboratory tests that are easily performed in many centers [10]. In addition to this scoring system, the FG severity index (FGSI), the Uludag FG severity index, the simplified FG severity index (sFGSI), and the NUMUNE Fournier Score are used to estimate disease severity and mortality [11,12,13,14]. Although no single scoring system provides a definitive prognosis, different predictive scoring systems for mortality at the time of initial presentation are needed and can be beneficial in guiding clinicians’ decision-making processes. This retrospective cohort study aimed to evaluate the effectiveness of the newly developed Fournier’s gangrene mortality index (FGMI) for predicting disease-related mortality and compare its effectiveness with the LRINEC score and various other inflammatory indices.

## 2. Materials and Methods

The study included patients over 18 years of age who were diagnosed with FG and underwent debridement after necrosis was detected in the genital or perianal region during clinical examination at the general surgery clinics of three tertiary hospitals in Şanlıurfa province between 2014 and 2024. All patient data were obtained from the hospital information management system. Primary follow-up of the patients was conducted by the general surgery clinics, and consultations from urology and other relevant departments were requested, as needed. FG diagnosis was made based on anamnesis, physical examination, and abdominal computed tomography. Demographic information, systemic diseases, laboratory findings, number of debridements performed, length of hospital stay, and mortality status of all patients were evaluated.

The patients included in the study were divided into two groups: deceased (*n* = 20) and surviving (*n* = 149). The blood parameters used to calculate the LRINEC score, including C-reactive protein (CRP); white blood cell count; hemoglobin; and sodium, creatinine, and glucose levels, were recorded. Additionally, preoperative values for platelet count, albumin, neutrophil count, neutrophil percentage, lymphocyte count, lymphocyte percentage, monocyte count, monocyte percentage, eosinophil count, eosinophil percentage, neutrophil-to-lymphocyte ratio (NLR), platelet-to-lymphocyte ratio (PLR), monocyte-to-lymphocyte ratio (MLR), and CRP-to-albumin ratio (CRP/Alb) were evaluated. Furthermore, according to the LRINEC score, patients were categorized into three risk groups: low (LRINEC score ≤ 5), moderate (LRINEC score 6–7), and high (LRINEC score ≥ 8) [10].

Preoperative parameters and scores used in the FGMI are presented in Table 1. Based on the total score and risk assessment, <5 points were categorized as low-to-moderate mortality risk and ≥5 points as high mortality risk.

### 2.1. Ethical Approval

Ethical approval for the study was obtained from the Harran University Clinical Research Ethics Committee with the decision number HRÜ/24.09.34, dated 1 July 2024.

### 2.2. Statistical Analysis

Descriptive analyses were conducted to describe the general characteristics of the study population. Visual (probability plots, histograms) and analytical tests (Kolmogorov–Smirnov and Shapiro–Wilk tests) were used to determine whether the data were normally distributed. Variables that did not show normal distribution were expressed as median (25th–75th percentiles). Mann–Whitney U test was used to compare two independent nonparametric variables, Kruskal–Wallis test was used for comparisons of three independent nonparametric groups, and Chi-square tests were used to compare categorical variables between the two groups. Categorical variables were reported as numbers and percentages. Furthermore, a new scoring system was created using parameters that showed a clinically significant difference between the deceased and surviving groups. To determine the mortality-predictive cutoff value of the new scoring system developed for Fournier’s gangrene patients, receiver operating characteristic (ROC) analysis was performed, and the area under the curve (AUC) was calculated. Based on this cutoff value, sensitivity and specificity of the scoring system in predicting mortality were calculated. An odds ratio risk analysis for mortality was performed according to the calculated cutoff value and expressed with a 95% confidence interval. For the multivariate analysis, the possible factors identified with univariate analyses were further entered into the logistic regression analysis to determine independent predictors of patient outcome. Hosmer–Lemeshow goodness-of-fit statistics were used to assess model fit. Post hoc power analysis was used for a 2 × 2 contingency table where the total score was categorized as 4 and below and 5 and above, α = 0.05, *N* = 169, and the effect size (w) was 0.395. A *p*-value of <0.05 was considered statistically significant in all analyses. All analyses were conducted using SPSS statistical software (IBM SPSS Statistics, Version 22.0. Armonk, NY, USA: IBM Corp.).

## 3. Results

A total of 169 patients diagnosed with FG, 87 of whom were men (51.48%), were included in the study. The median age of all patients was 53 (range: 40–63 years) years; 20 patients (11.8%) died. The median age was 64.50 years (range: 56–80 years) in the deceased group and 51 years (range: 39–60 years) in the surviving group. A significant difference was observed between the groups in terms of age (*p* < 0.001). The median length of hospital stay for the patients was 14 days (range: 9–22 days).

Although the number of debridements in the deceased group was lower compared to the surviving group, no significant difference was observed between the groups (*p* = 0.4). The presence of comorbidities was higher in the deceased group (75%) compared to the surviving group (57.05%), but this difference was not statistically significant (*p* = 0.1) (Table 2).

In the cultures taken from the 95 patients (56.21%), at least one bacterium was isolated. The most frequently isolated bacteria were *E. coli* (*n* = 53, 43.09%), *S. anginosus* (*n* = 11, 8.94%), and *K. pneumoniae* (*n* = 11, 8.94%) (Table 3).

There was no statistically significant difference in the LRINEC score between the deceased and surviving groups (*p* = 0.5). CRP levels were higher in the deceased group (29.47 mg/L) compared to the surviving group (20.76 mg/L), but this difference was barely within the significance limit (*p* = 0.05). In the deceased group, neutrophil count, neutrophil percentage, NLR, PLR, and CRP-to-Alb ratio were higher, whereas lymphocyte count, lymphocyte percentage, eosinophil count, eosinophil percentage, monocyte count, and monocyte percentage were lower compared to the surviving group. These differences were statistically significant (Table 4).

The multivariate analysis identified albumin (RR: 0.182, 95% CI: 1.106–27.027, *p* = 0.037) and NLR (RR: 0.055, 95% CI: 1.199–250.0, *p* = 0.037) as significant independent predictors of patient outcome. Other variables, such as age, creatinine, and lymphocyte percentage, were not statistically significant.

**Figure 1:**
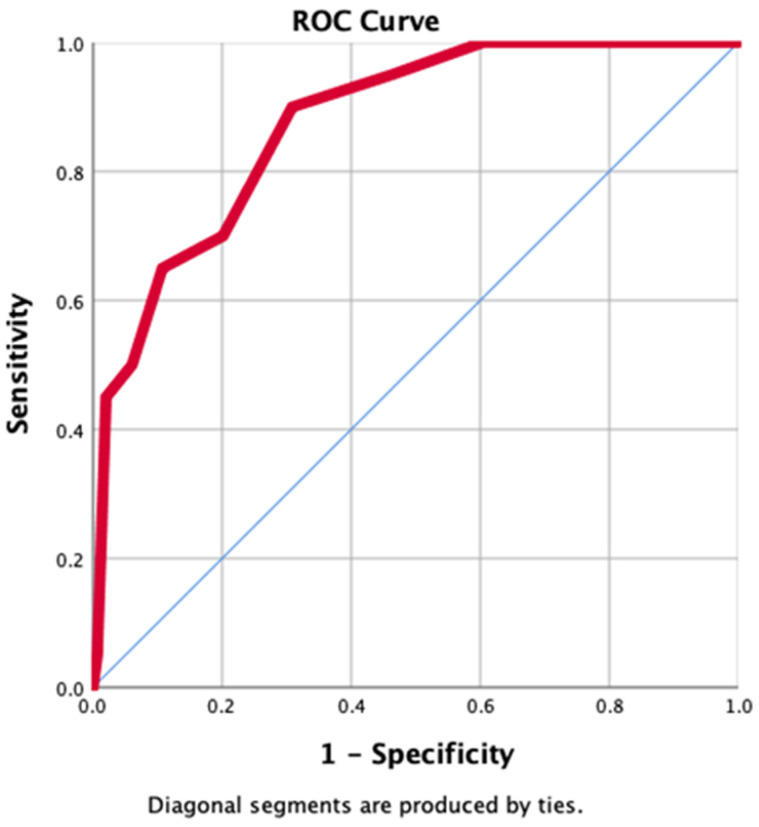
ROC curve predicting mortality in FG with FGMI score ≥ 5.

A ROC analysis was performed to determine the cutoff value for predicting mortality using the newly developed FGMI. According to the analysis, a score of 5 or higher had a ROC-AUC of 0.88 (95% CI: 0.80–0.95) with a sensitivity of 90% and specificity of 70% for predicting mortality (*p* < 0.001), (Figure 1). When univariate risk analysis was performed, the odds ratio revealed that mortality risk was 20 (4.48–90.91) times higher in patients followed up for FG with an FGMI score of ≥5 (*p* < 0.001). The post hoc power analysis result was calculated as 0.99925 (λ = 26.37; critical χ^2^ = 3.84).

## 4. Discussion

This study aimed to develop a new scoring system for predicting mortality in patients diagnosed with FG as well as to investigate the relationship between mortality and various inflammatory indices, particularly the LRINEC score. Although no significant relationship was observed between the LRINEC score and increased mortality, higher neutrophil counts, neutrophil percentages, NLRs, PLRs, and CRP/Alb ratios, and lower lymphocyte counts, lymphocyte percentages, eosinophil counts, eosinophil percentages, monocyte counts, and monocyte percentages in deceased patients suggest that these parameters are more significant indicators for predicting mortality. The high sensitivity (90%) and a 20-fold increase in mortality risk for the newly developed FGMI score with a cutoff value of ≥5 underscores its usefulness and significance as a scoring system in predicting mortality.

Data in the literature on which gender FG is more common indicate that the disease is much more common in the male gender [7,12,13,15]. In the present study, the rates of female patients were found to be close to the rates of male patients. The high birth rates in the region where we conducted our study, the fact that these patients mostly live in rural areas and have poor hygiene conditions pose a risk for FG. In addition, it was observed in our study that 56 (56%) of 100 patients with concomitant diseases such as DM were female and 44 (44%) were male. Our region is different from other regions in this respect. In addition, patients who applied to the general surgery clinic were included in our study, not patients who applied to the urology clinic. We attribute the higher number of female patients compared to other studies to these reasons.

Similar to other necrotizing soft tissue infections, older patients are more frequently affected by FG today [16]. In the study by Yilmazlar et al. [12], the age of 60 years and above was identified as an independent risk factor for mortality in FG patients. Benjelloun et al. [15] conducted a study in Morocco and discovered that patients who succumbed to FG (57.5 ± 19.24 years) were older compared to survivors (44.36 ± 16.05 years), with a statistically significant difference between the two groups (*p* < 0.001). In the present study, the median age of the deceased (64.50 years) was higher than that of the survivors (51 years), and this difference was statistically significant (*p* < 0.001). This finding indicates that age has a significant impact on mortality in patients diagnosed with FG.

Despite studies aimed at elucidating the etiology and pathophysiology of FG, mortality rates remain high [17]. Since the 19th century, epidemiological studies on necrotizing skin diseases have provided better predictions of the causes of FG and the associated mortality rates [9]. Various studies have shown that FG-related mortality is higher in the presence of comorbidities such as liver failure, chronic alcoholism, DM, and advanced age [7,18,19,20]. Mortality rates related to FG have been reported to range between 5% and 42% in various studies conducted in different geographical regions worldwide and in Turkey [1,12,16,21,22,23,24,25,26,27]. In the present study, the mortality rate was 11.8%, consistent with the literature. Although patients who died from FG had higher comorbidity rates compared to survivors, the difference between the groups was not statistically significant. This suggests that comorbidities may affect mortality, but this effect might be limited.

Predicting the course of the disease can be challenging for clinicians, but prognostic indicators such as FGSI, LRINEC, and NLR have been used in various studies to determine the severity and prognosis of FG [10,11,28,29]. The LRINEC score, developed by Wong et al. [10], is considered a strong diagnostic tool for necrotizing fasciitis based on various laboratory parameters, with scores above 6 indicating high positive predictive value. However, a systematic review investigating the reliability of the LRINEC score found a wide sensitivity range (43.2–80%), and its positive predictive (57–64%) and negative predictive values (42–86%) were lower than the initial results obtained by Wong et al. [10]. The authors recommended that the LRINEC score should be used concurrently with clinical evaluation and radiological diagnostic modalities for more accurate results [30]. In the study by Atilla et al. [26], no statistical difference was found between an LRINEC score of >6 and mortality. Similarly, Hahn et al. [21] showed no significant relationship between LRINEC (*p* = 0.7) and FGSI (*p* = 0.1) scores and mortality. In another case series, no significant relationship was observed between mean FGSI, LRINEC and NLR scores and mortality [28]. In contrast, Kincius et al. [22] showed that a 1-point increase in LRINEC score increased the risk of death 7.7-fold, and that the LRINEC cutoff value of 9 at initial presentation had a high predictive value for mortality. Similarly, in the study by Özlülerden et al. [24], the LRINEC score was identified as one of the predictive parameters for FG-related mortality. In the present study, it was shown that the LRINEC score was insufficient in predicting mortality. Our analyses revealed that, although the LRINEC score is a useful tool for supporting the diagnosis of necrotizing fasciitis, it is inadequate in accurately predicting the risk of death. This finding highlights the limitations of the LRINEC score, suggesting that it should not be solely relied upon in the management of patients with necrotizing fasciitis.

Laboratory findings of FG are nonspecific. Hematological and biochemical abnormalities such as anemia, thrombocytopenia, leukocytosis, hypokalemia, hyponatremia, hypocalcemia, hyperglycemia, elevated creatinine, azotemia, and hypoalbuminemia can be observed throughout the course of the disease [31]. Moreover, several studies have shown notable differences in laboratory parameters between patients who died from FG and those who survived. In the study by Kincius et al. [22], CRP levels were significantly higher in patients who died (*p* = 0.005) [22]. In the study by Atilla et al. [26], hemoglobin, platelet count, and serum sodium levels were lower, and creatinine levels were higher in deceased patients, although there was no significant difference in CRP levels between deceased and surviving patients. Kabay et al. [32] found that white blood cell count, blood urea nitrogen, and creatinine levels were higher in those who died from FG, and hematocrit, sodium, and albumin levels were lower. In the study by Özlülerden et al. [24], higher NLR levels were observed in deceased patients, with a cutoff value of 8.70 predicting mortality with 72.2% sensitivity and 52.3% specificity. Yim et al. [33] demonstrated that elevated NLR and PLR were more effective predictors of mortality in FG patients compared to the FGSI score. In the study by Demir et al. [34], hemoglobin and platelet counts were lower at initial presentation in patients who died from FG, whereas no statistically significant difference in NLR and white blood cell count was found between survivors and deceased patients. In a case series conducted in Indonesia, it was reported that NLR and FGSI had no predictive significance for FG-related mortality [35]. However, another study conducted in Indonesia reported that NLR had predictive significance for FG prognosis, and PLR did not [36]. In the present study, CRP levels were higher in deceased patients compared to survivors, although this difference was borderline significant (*p* = 0.05). As CRP reflects the presence and severity of infection, elevated CRP levels may be associated with mortality. Neutrophil count, neutrophil percentage, NLR, PLR, and CRP/Alb ratios were statistically higher in deceased patients, while lymphocyte count, lymphocyte percentage, eosinophil count, eosinophil percentage, monocyte count, and monocyte percentage were statistically lower compared to survivors.

The suboptimal sensitivity and specificity of existing scoring systems related to necrotizing fasciitis in predicting mortality prompted the development of a new scoring system, leading to the present study. By using hematological and biochemical parameters with predictive significance along with patient age at the time of initial presentation, a new scoring system—FGMI—was developed to distinguish between survivors and nonsurvivors. Age, creatinine, albumin, lymphocyte percentage, and NLR were identified as predictive parameters for mortality. Accordingly, for an FGMI cutoff value of ≥5, the sensitivity and specificity were 90% and 70%, respectively (*p* < 0.001). Another significant finding was that when the cutoff value was set at ≥5, it indicated a 20-fold increase in the risk of mortality. These results indicate the high predictive power of the FGMI score in terms of mortality and demonstrate its usefulness as a scoring tool.

### Limitations

This study has several limitations. First, its retrospective design imposes limitations on data quality and completeness. The study was limited to data from three hospitals in a specific geographical region, making it difficult to generalize the results to different populations or geographical regions. All patients diagnosed with FG were included in the study. Therefore, there is no bias, especially in terms of gender. Although 169 patients were included in the study, the relatively low number of patients who died (*n* = 20) limits the power of the statistical analyses and affects the reliability of some results. Additionally, this study did not directly compare FGMI’s effectiveness with other scoring systems, making it difficult to completely assess FGMI’s advantages or weaknesses over existing systems. The collection of laboratory and clinical data from different hospitals may have led to measurement errors or inconsistencies. Lastly, the absence of prospective studies to validate FGMI’s effectiveness and reliability raises questions about its potential effectiveness in clinical practice. These limitations suggest that the findings should be interpreted with caution and highlight the need for larger, prospective studies in the future.

## 5. Conclusions

The results obtained in the present study demonstrate that the FGMI score is a successful predictor of mortality that can be used at the initial clinical presentation of patients with FG. Another important finding is that the LRINEC score was not sufficiently effective in predicting mortality. These results underscore the importance of considering these parameters in clinical management. Furthermore, early intervention and close follow-up in high-risk patients are critical for reducing mortality rates.

## Figures and Tables

**Table 1:** Scoring of the Fournier’s gangrene mortality index.

Parameters	Units	Score
Age	≥60	2
	40–59	1
	≤39	0
Creatinine (mg/dL)	≥1.5	2
	1.0–1.49	1
	≤0.99	0
Albumin (g/dL)	≤2.5	2
	2.6–3.0	1
	≥3.1	0
Lymphocyte %	≤5%	2
	6–9%	1
	≥10%	0
NLR	≥15	2
	10–14	1
	≤9	0

NLR: Neutrophil-to-lymphocyte ratio.

**Table 2:** Demographic characteristics, comorbidity status, and general microbiological features of the patients.

Variable		Deceased (*n* = 20)	Surviving (*n* = 149)	Total (*n* = 169)	*p*
Age, year		64.50 (56–80)	51 (39–60)	53 (40–63)	<0.001
Gender	Male	9 (45%)	78 (52.35%)	87 (51.48%)	=0.4
	Female	11 (55%)	71 (47.65%)	82 (48.52%)	
Length of hospital stay, days		10.50 (3–22.5)	14 (9–22)	14 (9–22)	=0.3
Number of debridements		1 (1–3)	2 (1–3)	2 (1–3)	=0.4
Comorbidity	Yes	15 (75%)	85 (57.05%)	100 (59.17%)	=0.1
	No	5 (25%)	64 (42.95%)	69 (40.83%)	
Polymicrobial infection	Yes	5 (25%)	46 (30.87%)	51 (30.18%)	=0.4
	No	15 (75%)	103 (69.13%)	118 (69.82%)	

**Table 3:** Distribution of microorganisms isolated from patient cultures.

Microorganisms	*n*	%	Microorganisms	*n*	%
*E. coli*	53	43.09	*S. agalactiae*	4	3.25
*S. anginosus*	11	8.94	*A. baumannii* complex	4	3.25
*K. pneumoniae*	11	8.94	Other streptococci	2	1.63
*P. mirabilis*	10	8.13	*M. morganii*	2	1.63
*E. faecalis*	8	6.50	Other bacteria	5	4.07
*S. aureus*	7	5.69	Total	123	100
Other staphylococci	6	4.88			

**Table 4:** Comparison of preoperative laboratory parameters between the deceased and surviving groups.

Variables		Deceased (*n* = 20)	Surviving (*n* = 149)	Total (*n* = 169)	*p*
LRINEC	Low	5 (25%)	57 (38.26%)	62 (36.69%)	=0.5
	Moderate	3 (15%)	21 (14.09%)	24 (14.20%)	
	High	12 (60%)	71 (47.65%)	83 (49.11%)	
CRP (mg/dL)		29.47 (14.91–37.55)	20.76 (9.90–30.50)	21.98 (12.20–31.22)	=0.05
White sphere (10 × 10^3^/µL)		19.49 (14.80–24.27)	16 (11.60–20.40)	16 (11.69–20.80)	=0.1
Hemoglobin (g/dL)		11.25 (8.88–12.80)	11.80 (10.30–13.20)	11.70 (10.10–13.20)	=0.3
Sodium (mmol/L)		131 (128–134.50)	134 (130–138)	134 (130–137)	=0.2
Creatinine (mg/dL)		1.50 (1–2.01)	0.90 (0.70–1.17)	0.90 (0.71–1.31)	0.001
Glucose mg/dL		170.50 (106–263.50)	168 (106–338)	168 (106–336)	=0.9
Platelets (10 × 10^3^/µL)		271 (186–380.50)	301 (238–395)	299 (228–392)	=0.4
Albumin (g/dL)		2.68 (2.35–3)	3.20 (2.75–3.68)	3.10 (2.65–3.60)	0.001
Neutrophils (10 × 10^3^/µL)		15.75 (13.26–22.20)	13.53 (8.50–17.29)	13.69 (9.06–18.20)	0.042
Neutrophil %		89.85 (84.77–91.62)	82.50 (74.51–86.74)	83.50 (75.59–87.30)	<0.001
Lymphocytes (10 × 10^3^/µL)		0.83 (0.48–1.17)	1.60 (1.09–2.27)	1.49 (0.94–2.17)	<0.001
Lymphocyte %		4.81 (3.80–6.28)	9.56 (6.99–15.32)	8.83 (6.11–14.74)	<0.001
Monocytes (10 × 10^3^/µL)		0.60 (0.44–1.07)	0.96 (0.69–1.35)	0.90 (0.61–1.33)	0.023
Monocyte %		3.91 (2.39–4.84)	6.30 (4.80–8.39)	6.03 (4.53–8.15)	<0.001
Eosinophils (10 × 10^3^/µL)		0.01 (0–0.03)	0.05 (0.01–0.12)	0.04 (0.01–0.10)	<0.001
Eosinophil %		0.04 (0.02–0.21)	0.37 (0.10–1.14)	0.30 (0.06–1.00)	<0.001
NLR		18.21 (14.24–24.56)	8.55 (4.73–12.47)	9.59 (5.27–14.29)	<0.001
MLR		0.86 (0.45–1.29)	0.65 (0.37–0.97)	0.68 (0.38–0.97)	=0.1
PLR		371.70 (227.27–532.79)	185.33 (132.98–297.59)	192.76 (139.52–316.16)	<0.001
CRP/Alb		11.19 (6.28–13.75)	7.15 (2.77–9.28)	7.39 (3.72–10.64)	0.003

LRINEC: Laboratory risk indicator for necrotizing fasciitis, CRP: C-reactive protein, NLR: Neutrophil-to-lymphocyte ratio, PLR: Platelet-to-lymphocyte ratio, MLR: Monocyte-to-lymphocyte ratio, CRP/Alb: CRP/albumin.

## Data Availability

Raw data that support the findings of this study are available from the corresponding author upon reasonable request.
